# An Environmentally Friendly Inverse Microemulsion Method to Synthesize Polyacrylamide

**DOI:** 10.3390/ma15175927

**Published:** 2022-08-27

**Authors:** Qing Guo, Longlong Yin, Xiao Wang, Jing Yuan, Qianfeng Zhang

**Affiliations:** 1 Institute of Molecular Engineering and Applied Chemistry, Anhui University of Technology, Ma’anshan 243002, China; 2 Department of Civil Engineering, Tongling University, Tongling 244000, China; 3 Donadeo Innovation Centre of Engineering, University of Alberta, 9211-116 Street NW, Edmonton, AB T6G 1H9, Canada

**Keywords:** inverse emulsion, electrical conductivity, hydrophile–lipophile balance (HLB) value

## Abstract

Polyacrylamide (PAM) was prepared by a new method of inverse microemulsion polymerization, with (NH_4_)_2_S_2_O_8_-Na_2_SO_3_ as initiator and liquid paraffin/Span80-Op10/AM-H_2_O-NaAc as polymerization system in this paper. The effects of initiator dosage, emulsifier dosage, monomer concentration, oil–water ratio, and temperature on molecular weight, electrical conductivity, particle size distribution, and monomer conversion were studied as well. The results indicate that that the more stable Polyacrylamide (PAM) polymer was prepared under the conditions of initiator dosage of 0.4~0.5%, emulsifier dosage of 55~60%, temperature of 40~45 °C, hydrophile–lipophile balance (HLB) value of 8.0~8.2, and NaAc concentration of 3%.

## 1. Introduction

Polyacrylamide (PAM) is a linear polymer, and its main chain is with many amide groups. These groups can be modified to make many polyacrylamide derivatives, the products of which are widely used in many fields [1,2,3].

Traditional emulsion polymerization usually includes monomer, aqueous phase, water-soluble initiator, and emulsifier (or stabilizer) [4]. In recent years, emulsion polymerization has become a research hotspot and plays an important role in many industrial applications, such as coatings [5,6,7,8], adhesives [9,10], and coatings [11]. However, the stability of emulsion polymerization is a key issue. Microemulsion is a semi-transparent, heterogeneous, dynamically quasi-stable system that contains two immiscible fluids, usually water and oil, as well as surfactants [12]. In addition, reverse-phase microemulsion (W/O) is a mixture composed of water, oil, surfactant, and cosurfactant, which can be regarded as a kinetically quasi-stable and translucent system [13,14]. Microemulsion polymerization [15,16,17,18] is the nucleation of liquid droplets through monomer, that is, the monomer droplets are directly transformed into polymer particles (different from emulsion polymerization), and the general particle size range is 50–500 nm. In conclusion, the microemulsion is a semi-transparent heterogeneous kinetic quasi-stable system containing two insoluble aqueous and oil phases [19,20,21]. Therefore, microemulsion polymerization has been paid more and more attention and is widely used in the industry. However, microemulsions and microemulsion polymerization require a large amount of surfactant. Due to the high cost of surfactants and post-treatment to remove surfactants after polymerization, this drawback has hindered the large-scale development of the process. In order to make microemulsion polymerization more practical, the amount of surfactant should be reduced as much as possible, and the content of polymer should be increased as much as possible [22]. Microemulsion polymerization has proven to be a promising nanoemulsion synthesis technology [23,24], which has great industrial application value. However, too much oil phase reduces the solid phase content and increases the production cost. Therefore, copolymers with high solid content and large molecular weight were obtained under the condition of small oil–water ratio. Microgels prepared by reverse microemulsion polymerization have the advantages of small particle size and narrow particle size distribution [25,26]. In recent years, reverse-phase microemulsion polymerization has attracted much attention and become an increasingly important research field in the synthesis of water-soluble polymers. Polyacrylamide was prepared by reverse-phase microemulsion polymerization, and the properties of microemulsion polymerization were verified. In order to achieve substance stability and industrial production, it is generally necessary to synthesize substances with nanoscale particles, and such nanoscale polymer particles are mainly synthesized by microemulsion polymerization [27]. Hydrophobic modified polyacrylamide nanoemulsion was synthesized by copolymerization of acrylamide monomer and new polymerizable surfactant by reverse-phase microemulsion copolymerization technology, which has a good application effect in improving oil recovery [28].

Generally speaking, there are three kinds of conventional preparation methods: conventional emulsion polymerization, microemulsion polymerization and semi-batch emulsion polymerization. The particle size of polymers prepared by conventional emulsion polymerization is generally above 100 nm, while the particle size of polymers prepared by microemulsion polymerization is generally 10–50 nm, which is much smaller than that of polymers prepared by conventional emulsion polymerization [29]. Due to the emulsion polymerization system instability, opaque, cause the turbidity and precipitate for a long time, the microemulsion polymerization system can be translucent, dynamics of metastable system, inverse microemulsion preparation of microemulsion has good dispersibility and micelle size [30,31,32] with the advantage of being easy to control. The existence of the emulsifying agent (surfactant) makes its viscosity increases, and easy phase separation has not occurred. Therefore, reverse phase microemulsion method was chosen to synthesize the polymer.

In this study, the influence of various factors on the synthesis of stable polyacrylamide microemulsions was discussed, and the instability and turbidity of the products prepared by traditional emulsions were overcome. At the same time, polyacrylamide (PAM) was prepared by a new method of inverse microemulsion polymerization with (NH_4_)_2_S_2_O_8_-Na_2_SO_3_ as initiator and liquid paraffin/Span80-Op10/AM-H_2_O-NaAc as polymerization system. The effects of initiator dosage, emulsifier dosage, monomer concentration, oil–water ratio, and temperature on polymerization system were studied.

## 2. Experiment

### 2.1. Experimental Materials

Experimental materials such as Span80 (sorbitol oleate) (Beijing Yili Chemical Products Co., Ltd., Beijing, China), Op10 (alkyl phenol polyoxyethylene ether) (Beijing Yili Chemical Products Co., Ltd.), liquid paraffin (Beijing Yili Chemical Products Co., Ltd.), acrylamide (Beijing Yili Chemical Products Co., Ltd.), ammonium persulfate (Tianjin Damao Chemical Reagent Factory, Tianjin, China), sodium acetate (Beijing Yili Chemical Products Co., Ltd.), sodium sulfite (Tianjin Damao Chemical Reagent Factory), sodium chloride (Beijing Century Red Star Chemical Co., Ltd., Beijing, China), sodium thiosulfate (Liaoning Quanrui Reagent Co., Ltd., Jinzhou, China), potassium bromate (Liaoning Quanrui Reagent Co., Ltd.), soluble starch (Liaoning Quanrui Reagent Co., Ltd.), hydrochloric acid (Liaoning Quanrui Reagent Co., Ltd.), anhydrous sodium carbonate (Liaoning Quanrui Reagent Co., Ltd.), and anhydrous ethanol (Liaoning Quanrui Reagent Co., Ltd.) will be used in this study.

### 2.2. Experimental Procedures

**Figure 1:**
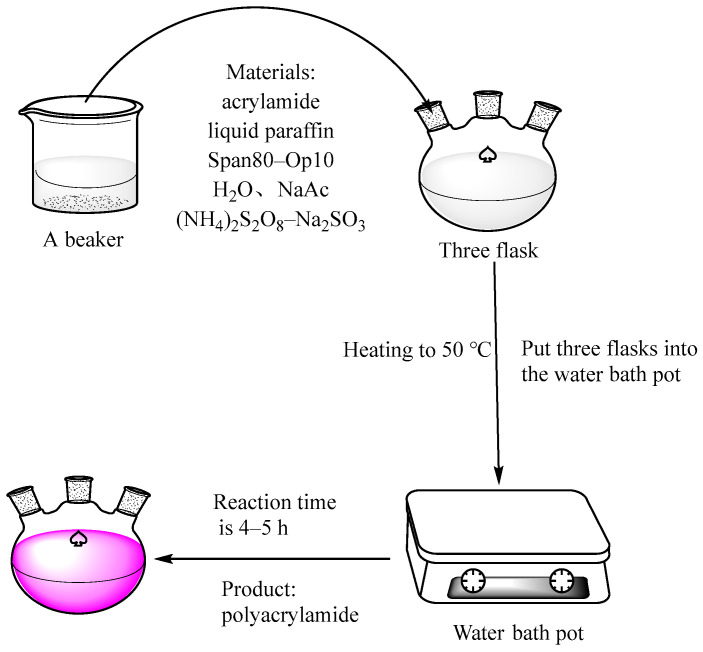
Diagram of the experimental setups and procedures.

A certain amount of paraffin and emulsifier was added to a 500 mL three-mouth flask, and then the pre-prepared acrylamide aqueous solution was added after stirring for 15 min. The reverse-phase microemulsion was prepared at a rotational speed of 800 R/min, as shown in Figure 1. The constant temperature water bath was controlled to 50 °C, and a certain concentration of initiator was added to initiate the reaction. The reaction was continued at 50 °C for 4 h, and the stirring was stopped to end the reaction.

### 2.3. Determination of Conversion Degree

In the determination of monomer conversion, first prepare sodium thiosulfate standard solution C (Na_2_S_2_O_3_·5H_2_O = 0.1 mol/L), starch indicator, and bromine reagent. The next steps were as follows: first, titration of blank sample was carried out, 10 mL distilled water, 25 mL bromine reagent and 10 mL of 1:1 hydrochloric acid were put into iodine measuring bottle, the lid was closed and sealed with KI solution. After the reaction, open the plug, add 10 mL of 20% KI solution, close the lid, seal with KI solution, place in the dark for 20 min, titrate with 0.1 mol/L sodium thiosulfate solution, and add 1% starch reagent. Next, the sample titration was carried out. Firstly, 10 g sample was taken, and 50 mL ethanol was added to settle overnight for filtration. The weight of the filtered supernatant was m, 1 g of the filtered supernatant was taken, and 50 mL water was added for constant volume. Open the plug, add 10 mL of 20% KI solution, close the lid, seal with KI solution, place in the dark for 20 min, titrate with 0.1 mol/L sodium thiosulfate solution, and add 1% starch reagent. Finally, the monomer conversion rate is calculated by using the following Equation (1), however, Equation (1) represents the residual monomer acrylamide content in polyacrylamide, that is, the percentage of monomer that is not transformed, and then the percentage of monomer that is not involved in the reaction is used to obtain the conversion rate of monomer.

(1) $$AM\%=\frac{\frac{1}{2}(V1-V2)\cdot C\cdot m}{10\times 1\times Solidcontent}$$

Equation (1): *AM*%—the content of acrylamide; *V*1—Volume of sodium thiosulfate standard solution consumed by blank titration, mL; *V*2—Volume of sodium thiosulfate standard solution consumed for titration of the sample, mL; *C*—Sodium thiosulfate standard solution concentration, mol/L; *m*—Ethanol sedimentation overnight filtration weighing liquid mass, g.

### 2.4. Determination of Intrinsic Viscosity

Using 1.0 mol/L sodium chloride solution as solvent, the polymer solution with concentration of 2000 ppm was prepared. The time of solvent and polymer solution flowing through the two scales of the Viscosimeter was measured with an aerated suspension column at a temperature of 30 °C. The intrinsic viscosity of the polymer can be calculated from the measured values.

### 2.5. Determination of Apparent Viscosity

Brookfield DV- ⅱ viscometer (rotor 0^#^) was used. The experimental viscosity was measured at 45 °C and the shear rate was 6 S^−1^. First, prepare the sample solution to be tested, weigh 0.1 g of sodium chloride, and add it to the polymer sample solution for dissolution, and then measure the sample solution to be tested. First, raise the temperature of the viscometer to 45 degrees Celsius, put the rotor into it, put the solution to be tested under the small head of the rotor, operate as instructed, and record the data.

### 2.6. Calculation of HLB Value

Since the HLB values of non-ionic surfactants used in this paper are additive, the following Equation (2) can be used to calculate the HLB values of two or more surfactants mixed. For example, the HLB value calculated by mixing two surfactants is 8.36 after mixing Span80 (HLB = 4.3) and Op10 (HLB = 14.5). In other words, the HLB value can be obtained by weighing 30.1 g of Span80 and 19.9 g of Op10.

(2) $$HLB_{12}=\frac{HLB_{1}\;m_{1}+HLB_{2}\;m_{2}}{m_{1}+m_{2}}$$

Equation (2): *m*_1_ and *m*_2_—represent the mass of surfactants 1 and 2, respectively; *HLB*_1_ and *HLB*_2_ represent the HLB values of surfactants 1 and 2, respectively; *HLB*_12_—HLB value after mixing of two surfactants.

## 3. Results and Discussion

The influence of each component on the microemulsion system of acrylic acid was studied, the propylammonium acrylate reverse microemulsion system was optimized, and a stable reverse microemulsion with low emulsifier dosage and high monomer content was obtained.

The structure type of microemulsion system can be determined by measuring its electrical conductivity. The stability of the system and the amount of solubilizing water can be roughly seen from the fluctuation of conductivity with the ruler phase.

Electrical conductivity is one of the commonly used methods to study microemulsions. During the polymerization of microemulsion system, the change trend of electrical conductivity can be divided into four stages [33,34]: the first stage is a slow increase of electrical conductivity, which is permeated due to the aggregation of reverse microemulsion droplets, at this time, W/O microemulsion system, its outer oil is slightly conductive or non-conductive. In the second stage, the conductivity increases linearly rapidly, and the microemulsions droplets fuse with each other due to the sticky collisions. This is because when the concentration of water droplets in W/O type microemulsion is high enough, the droplets collide with each other in a sticky collisions way, and the direct result of such sticky collisions is the formation of many narrow and small water pipes in the oil continuous phase. The third stage, the increase and slow conductivity, is due to the formation of a double continuous phase structure. At this time, W/O and O/W microemulsions co-exist, but they are not spherical. Small water pipes are interconnected so that the whole system will form a network of water pipes in the oil phase and oil pipes in the water phase, which are interlaced with each other. This is a double continuous phase. At the last stage, the electrical conductivity will increase linearly due to the formation of oil-in-water microemulsion, and also due to the inorganic ions contained in the water phase. Table 1 shows similar rules and observes the emulsion state after each group of orthogonal experiments stood for 7 days, laying a foundation for further experimental optimization.

**Figure 2:**
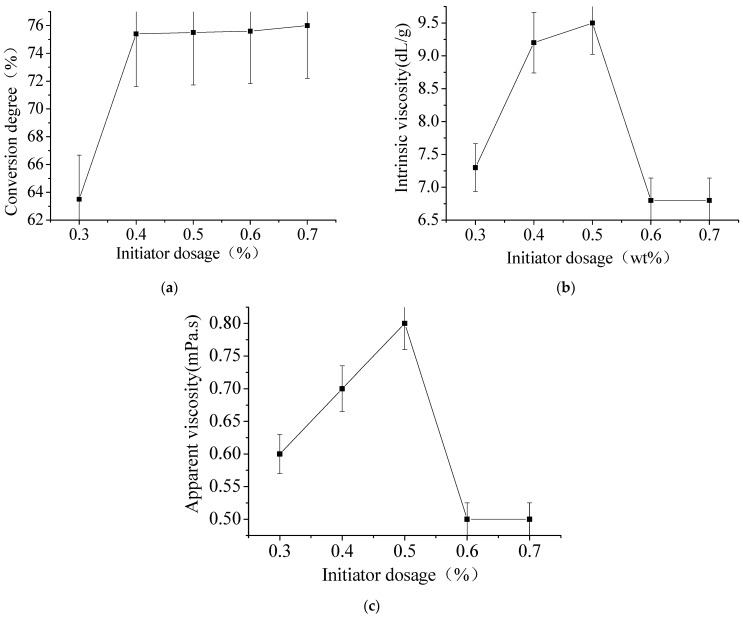
The effect of initiator dosage. (**a**) Effect of initiator dosage on conversion degree. (**b**) Effect of initiator dosage on intrinsic viscosity. (**c**) Effect of initiator dosage on apparent viscosity.

It can be seen from Table 1 that the microemulsion is very stable over the initiator dosage range. As can be seen from Figure 2a, when the initiator dosage reaches 0.4%, the polymerization conversion degree reaches 75.5%. This is because when the amount of initiator increases, the number of active centers that can trigger monomer polymerization increases and the conversion degree of polymerization reaction increases. However, when the amount of initiator reaches a certain value, the effective collision frequency between molecules does not increase significantly, so the conversion degree of polymerization reaction does not change much. Furthermore, in Figure 2b, the intrinsic viscosity of copolymer first increases and then decreases with the concentration of initiator. Because the large number of microdroplets in the inverse microemulsion system, the possibility of the second free radical entering the microdroplets is small. According to Figure 2c, the initiator dosage increases, the molecular weight increases, and the viscosity of the system also increases. Therefore, the presence of initiator as electrolyte increases the particle size, but also reduces the viscosity of the system.

**Figure 3:**
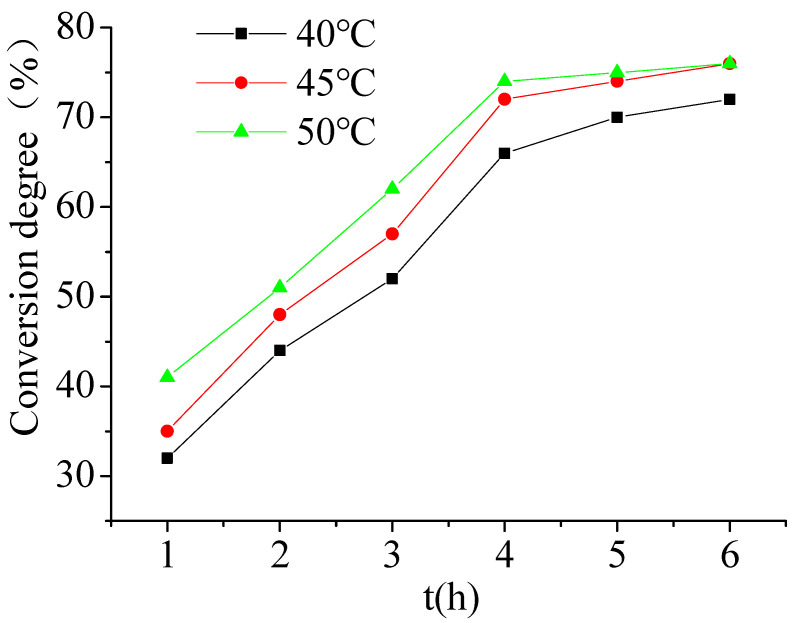
The effect of temperature.

Figure 3 shows that on the one hand, the influence of reaction time on the polymerization conversion and the start of the polymerization reaction of 3–4 h, along with the increase of reaction time and the conversion degree, increased significantly. This is because the emulsifier in the water phase and the decomposition generates free radicals caused by water phase polymerization of the monomer. Chain growth is fast, and latex particles formed. After about 4 h, the conversion degree reached about 70%, and the growth of the reaction conversion degree tends to be gentle, which may be because with the expansion of the diameter of the latex particle, the monomer inside the particle is blocked to the outer diffusion, so the conversion degree tends to be gentle with the increase of time. On the other hand, the conversion degree of polymerization reaction is affected by temperature. With the increase of temperature, the conversion degree of polymerization reaction also increases. This is because when the polymerization temperature is low, the decomposition of initiator, the diffusion rate of monomer and the polymerization rate are very small. With the increase of temperature, the decomposition rate of initiator, the diffusion rate of monomer, the effective collision between molecules, and the polymerization rate all increase.

**Figure 4:**
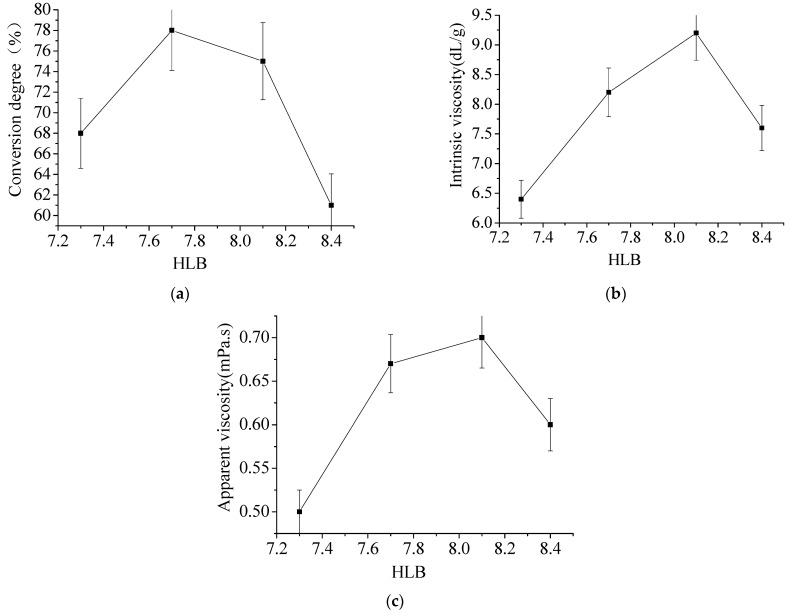
The effect of hydrophile–lipophile balance (HLB) value. (**a**) Effect of HLB value on conversion degree. (**b**) Effect of HLB value on intrinsic viscosity. (**c**) Effect of HLB value on apparent viscosity.

It can be seen from Figure 4a that when the HLB value is 7.7, the conversion degree of the polymerization reaction reaches the maximum. Then, as the HLB value increases, the conversion degree of the polymerization reaction begins to decrease. This is because with the increase of the oil phase content in the system, the number of water-phase monomers decreases, and the conversion degree of the monomers also decreases, so the conversion degree of the polymerization reaction decreases. Furthermore, Figure 4b reflect that the hydrophile–lipophile balance (HLB) value is related to the ratio of compound emulsifier, so we can discuss the influence of emulsifier on the intrinsic viscosity of polymer. Figure 4c infers that the conversion degree of the system increases and the concentration of the polymer system increases, so the apparent viscosity of the polymer also increases. However, when it reaches a certain level, the conversion degree, system permeability, and solubilization ability decrease, so the apparent viscosity of the polymer also decreases.

**Figure 5:**
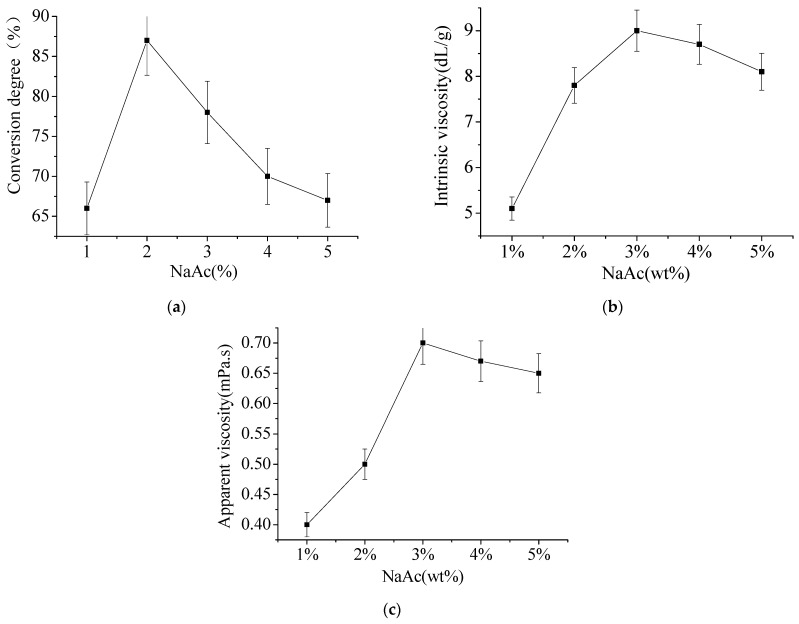
The effect of NaAc dosage. (**a**) Effect of NaAc dosage on conversion degree; (**b**) Effect of NaAc dosage on intrinsic viscosity; (**c**) Effect of NaAc dosage on apparent viscosity.

In microemulsion system, the phase transition can be promoted by increasing the concentration of aqueous salts. As can be seen from Figure 5a, with the increase of NaAc concentration, the conversion degree of acrylamide (AM) gradually increased to the maximum 87% and then decreased. This is because, with the increase of salt concentration, water phase microemulsion system Na^+^ on anionic surfactant had a greater influence on the formation of microemulsion, reducing the hydrophilic ionic surfactant. The salt concentration is low, with the increase of water phase salt concentration, capacity of the same amount of oil and water needed for the quality of the surfactant scoring significantly lower, enhancing the system’s expansion ability. The content of water phase increased, and the content of monomer that could participate in the reaction also increased, so the degree of monomer transformation increased, and the degree of polymerization reaction transformation also increased. However, with the further increase of salt concentration, salt will produce the effect of demulsification, making the system completely immiscible and stratified, and the degree of polymerization transformation will also decrease. As can be seen from Figure 5b, when NaAc concentration is low, the strong salting out electrolyte effect contributes to the stability of the microemulsion. When the concentration of NaAc is higher, it mainly plays the role of chain transfer. It can be seen from Figure 5c that salting out strong electrolyte salts can improve the stability of non-ionic microemulsion and adjust the matching of emulsifier with oil and water.

**Figure 6:**
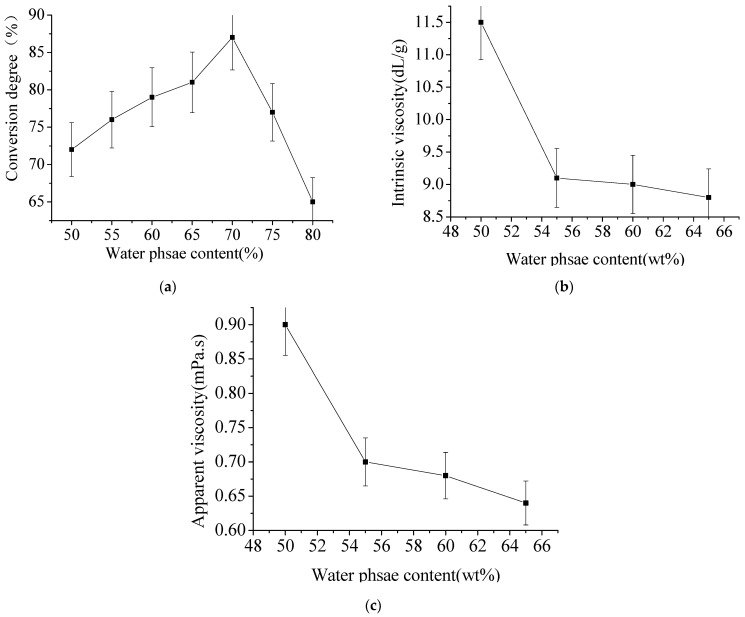
The effect of water phase content. (**a**) Effect of water phase content on conversion degree; (**b**) Effect of water phase content on intrinsic viscosity; (**c**) Effect of water phase content on apparent viscosity.

As shown in Figure 6a, the microemulsion is W/O system, the external phase is oil phase, and the conversion degree of polymerization reaction is low. With the increase of the content of water phase, the droplet converges and collides with each other, which increases the reaction degree and the conversion degree of monomer and polymerization reaction. However, as the water content continues to increase, the W/O microemulsion system will shift to O/W system. This system is neither the thermodynamic stability system nor the dynamic metastable system, lowering the monomer conversion degrees. At the same time, it may be due to the high content of water phase and the relatively low content of oil phase. More microemulsion particles are formed, and these particles will gather. The diameter of latex particles increases, and the limited collision frequency of monomer molecules decreases, so the conversion degree begins to decline. As shown in Figure 6b, with the increase of water phase content and the decrease of oil phase content, the system will form an unstable state, the monomer conversion degree will also decrease, and the intrinsic viscosity of the resulting polymer will also decrease, which conforms to the changing rule of inverting microemulsion conversion degree. Figure 6c infers that the system changes from transparent to translucent and forms a viscous liquid, causing the system to enter the liquid crystal region. In the liquid crystal region, due to the increase of water phase content, the core volume of inverted microemulsion expands continuously, which destroys the structure of inverted microemulsion.

**Figure 7:**
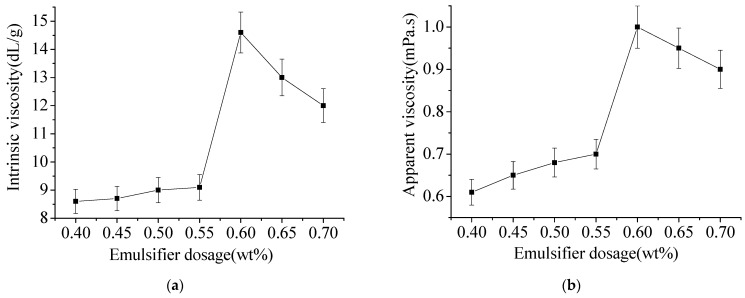
The effect of emulsifier dosage. (**a**) Effect of emulsifier dosage on intrinsic viscosity. (**b**) Effect of emulsifier dosage on apparent viscosity.

In Figure 7a, Polyacrylamide (PAM) intrinsic viscosity in the same oil–water ratio, the higher the concentration of emulsifier, polyacrylamide (PAM) intrinsic viscosity is smaller. The increase of the amount of emulsifier will strengthen the chain rotation effect of emulsifier in the polymerization, resulting in the reduction of the intrinsic viscosity of the polymer, and the high content of emulsifier cost increases. According to Figure 7b, the amount of emulsifier increases, the latex particles become smaller, resulting in the increase of the contact area between the particles, manifested as the rapid increase in the viscosity of latex. The main reason is that the increase of the amount of emulsifier makes the polymerization growth chain have more chances to transfer to the emulsifier and terminate, resulting in its decrease.

## 4. Conclusions

In conclusion, polyacrylamide (PAM) was prepared by reverse phase microemulsion polymerization with (NH_4_)_2_S_2_O_8_-Na_2_SO_3_ as initiator and liquid paraffin/Span80-Op10/AM-H_2_O-NaAc as polymerization system. The effects of initiator dosage, emulsifier dosage, monomer concentration, oil–water ratio, and temperature on polymerization system were studied. The results showed that the stable microemulsion system was obtained by controlling the initiator concentration in the range of 0.4~0.5%, the hydrophilic lipophilic equilibrium (HLB) value of 8.3, the emulsifier mass fraction of 50~55%, the reaction temperature of 45~50 °C, and the electrical conductivity. At the same time, a small amount of electrolyte salt NaAc can also improve the stability of the microemulsion system. In conclusion, this experimental study provides a theoretical basis for the preparation of stable polyacrylamide microemulsion.

## Figures and Tables

**Table 1:** Effect of electrical conductivity.

Sample	Electrical Conductivity (µs/cm)	7d	Sample	Electrical Conductivity (µs/cm)	7d	Sample	Electrical Conductivity (µs/cm)	7d
Initiator (0.3%)	0.021	Translucent Unstable	NaAc 1%	0.01	Translucent Unstable	Temperature 40 °C	0.009	Translucent Unstable
Initiator (0.4%)	0.023	Translucent Kinetically Stable	NaAc 2%	0.01	Translucent Unstable	Temperature 45 °C	0.069	Translucent Kinetically Stable
Initiator (0.5%)	0.024	Translucent Kinetically Stable	NaAc 3%	0.055	Translucent Kinetically Stable	Temperature 50 °C	0.011	Translucent Unstable
Initiator (0.6%)	0.029	Translucent Unstable	NaAc 4%	0.078	Translucent Kinetically Stable	-	-	-
Initiator (0.7%)	0.03	Translucent Unstable	NaAc 5%	0.0107	Translucent Unstable	-	-	-
**Sample**	**Electrical Conductivity (µs/cm)**	**7d**	**Sample**	**Electrical Conductivity (µs/cm)**	**7d**	**Sample**	**Electrical Conductivity (µs/cm)**	**7d**
HLB 8.36	0.021	Translucent Unstable	Water phase content 50%	0.017	Translucent Unstable	Liquid paraffin	0	Transparent Stable
HLB 8.05	0.023	Translucent Kinetically Stable	Water phase content 55%	0.025	Translucent Kinetically Stable	-	-	-
HLB 7.72	0.024	Translucent Unstable	Water phase content 60%	0.031	Translucent Kinetically Stable	-	-	-
HLB 7.30	0.029	Translucent Unstable	Water phase content 65%	0.047	Translucent Unstable	-	-	-
-	-	-	Water phase content 70%	0.014	Translucent Unstable	-	-	-
-	-	-	Water phase content 75%	0.01	Translucent Unstable	-	-	-

## Data Availability

All relevant data generated by the authors or analyzed during the study are within the paper.
